# Using Geographic Information Systems to Compare Municipal, County, and Commercial Parks Data

**DOI:** 10.5888/pcd10.120265

**Published:** 2013-06-06

**Authors:** Kelly R. Evenson, Fang Wen

**Affiliations:** Author Affiliations: Fang Wen, Gillings School of Global Public Health, Department of Epidemiology, University of North Carolina, Chapel Hill, Chapel Hill, North Carolina.

## Abstract

**Introduction:**

Parks are an integral part of a favorable built environment, and several studies have found a positive association between a favorable built environment and physical activity. Parks data are available to researchers from various sources; however, the accuracy of data sources in representing parks is unknown. This study compared secondary parks data obtained from a commercial vendor with data from municipal/county government records, all of which were verified by using Internet searches, telephone inquiries, or on-the-ground audits.

**Methods:**

We studied large metropolitan areas in 3 states: North Carolina (1,837 sq mi), Maryland (1,351 sq mi), and New York (260 sq mi). We collected information on park land area (shapefiles) from municipal/county governments from 2009 through 2012 and from a commercial source in 2010.

**Results:**

Commercial parks data did not include 31.1% (119/383, 20.3 sq mi) of North Carolina, 42.9% (187/436, 21.8 sq mi) of Maryland, and 71.7% (640/892, 13.5 sq mi) of New York parks that we found and verified from municipal/county sources. Municipal/county data did not include 15.7% (60/383, 9.9 sq mi) of North Carolina parks, 27.5% (120/436, 74.6) of Maryland parks, and 9.0% (80/892, 6.3 sq mi) of New York parks that we found and verified from commercial sources.

**Conclusion:**

In this study, the combination of commercial and municipal/county data sources that were verified provided the most complete and accurate shapefile. The quality of secondary sources of parks data should be checked prior to use and, if needed, methods incorporated to improve the capture of parks.

## Background

Numerous studies have found a positive association between a favorable built environment and physical activity, such as walking or bicycling (1–4). Parks are an integral part of the built environment. They exist in many communities and often provide free places for physical activity (5). Researchers and public health practitioners have studied access to parks to help plan where new parks should be developed, to identify underserved locations, and to determine what facilities should be offered at the parks (6–9). The development of geographic information systems (GIS) has facilitated the study of spatial access and use of parks.

Researchers using GIS to study parks can obtain parks data from several sources. Sources include using commercial sources (9,10), assembling park locations from local jurisdictions, such as municipal or county governments (7,11), and on-the-ground audits that include measuring park boundaries in the field (12–14). Each of these sources varies in cost and time required. To our knowledge, no study has compared the accuracy of commercial and municipal/county data sources in representing park geographic area and amenities. Our study compared parks data obtained from commercial sources with those from municipal and county government sources for 3 large metropolitan areas in 3 US states: North Carolina, Maryland, and New York. The findings highlight strengths and limitations of both data sources. We also explored the effect of parks being omitted from both data sources.

## Methods

We defined a park as a public place set aside for physical activity and enjoyment. This definition did not include cemeteries, mobile home parks, historic sites, professional stadiums, country clubs, zoos, private parks, private facilities (such as stand-alone baseball or tennis facilities), or stand-alone recreation centers.

### Study area

The study areas corresponded to 3 of 6 US locations from the Multi-Ethnic Study of Atherosclerosis (MESA), a cardiovascular cohort study that enrolled 6,814 participants from 2000 through 2002 (15). The 3 study areas, as defined by the MESA Study, were expanded to capture areas where participants had moved since enrollment. In this paper, we refer to the study areas by state: North Carolina (Davidson, Davie, Guilford, Forsyth, Randolph, Rockingham, Stokes, Surry, and Yadkin counties [1,837 sq mi]); Maryland (79 zip code areas in Anne Arundel, Carroll, Harford, Howard, and Baltimore counties and Baltimore city [1,351 sq mi]); and New York (183 zip code areas in Bronx, Brooklyn, Manhattan, and Queens boroughs, and Westchester County [260 sq mi]).

### Data collection

From 2009 through 2012, we used municipal or county GIS shapefiles (GIS files that include the park name and an outline of each park drawn as a polygon) to locate parks, most of which came from planning, parks, and recreation departments. In a few instances, we used Google maps (http://maps.google.com/maps) to draw a park boundary when no other outline of the park was available. If only part of the polygon for a confirmed park was in the study area, we included it in our study. Parks with multiple polygons but the same name were manually merged and designated as 1 park. Parks were verified by using Internet searches, telephone inquiries, and if necessary on-the-ground audits.

To determine the amenities available at each park (eg, tennis courts, basketball hoops, swimming pools), we searched online, contacted municipal/county departments, or visited the park. This process also allowed us to verify that the park conformed to our park definition.

We obtained commercial data on parks for 2010 from Esri (Esri, Redlands, California). Esri metadata (a summary document containing information on the data set) indicated that parks and forests were identified at the national, state, and local levels, including county and regional parks, and referenced Tele Atlas MultiNet North America (Lebanon, New Hampshire; www.teleatlas.com). We verified the existence of parks and park facilities that Esri identified by using the same methods we used to verify municipal/county sources, primarily through Internet searches and telephone inquiries.

### Statistical analysis

We used several tools in ArcGIS 10.0 (Esri, Redlands, California) to compare the park shapefiles obtained from the commercial sources with files obtained from municipal/county sources. For each of the 3 states, GIS files from both data sources were assembled and overlaid using the state plane coordinate system. Parks that partially overlapped were explored manually in ArcGIS by comparing the park name, shape, and percentage of the area overlapping to determine whether the parks were the same.

The area of each park polygon was calculated for both data sources by using the ArcGIS calculating geometry tool. With the 2 shapefiles projected on top of each other in ArcGIS, the concordant park area from the 2 data sources was extracted, corresponding to spatially matched areas. This area in square miles was calculated for both matched and mismatched park areas.

To quantify the impact of missed parks (defined as parks reported in one data source but not the other), we calculated an indicator described in the Centers for Disease Control and Prevention’s (CDC’s) recommended strategies to enhance or create access to places for physical activity (16). The indicator for the extent of the public’s access to parks was defined as “the percentage of US census blocks with parks.” The indicator was calculated as the proportion of 2010 census blocks that have at least 1 park within the block or within 0.5 miles of the block boundary. This metric was calculated for both data sources separately and for both combined. As a second metric to quantify the effect of missed parks, the percentage of parks with each type of facility missed (eg, basketball court, swimming pool) was calculated for both data sources.

## Results

Overall, we verified the existence of 383 parks in the NC study area, 436 parks in the Maryland study area, and 892 parks in the New York study area (Table 1). The commercial data source did not include the following percentage of parks found and verified in municipal/county sources: 31.1% (119/383, 20.3 sq mi) in North Carolina, 42.9% (187/436, 21.8 sq mi) in Maryland, and 71.7% (640/892, 13.5 sq mi) in New York. The municipal/county data sources did not include the following parks found and verified in the commercial source: 15.7% (60/383, 9.9 sq mi) in North Carolina, 27.5% (120/436, 74.6 sq mi) in Maryland, and 9.0% (80/892, 6.3 sq mi) in New York. Municipal/county data sources showed higher percentages of land area with parks for North Carolina and New York than did the commercial data sources but a lower percentage for Maryland.

**Table 1:** Comparison of Parks Data Obtained From Municipal/County Sources with Data Obtained from Commercial Sources in 3 Locations: North Carolina, Maryland, and New York, 2009–2012

Park Details	North Carolina^a^, n = 383^b^		Maryland^c^, n = 436^b^		New York^d^, n = 892^b^	
	Municipal/ County^e^	Commercial^f^	Municipal/ County^e^	Commercial^f^	Municipal/ County^e^	Commercial^f^
**Number of parks**						
Number of parks, total	323	261	316	246	812	251
Parks in both data sources^g^	204	201	129	126	172	171
Parks in municipal/county data but not in commercial data	119	NA	187	NA	640	NA
Parks in commercial data but not in municipal/county data^h^	NA	60	NA	120	NA	80
**Park area (sq mi)**						
Park area, total	43.0	32.5	67.9	120.7	31.8	24.7
Park area spatially overlaid^i^	22.7	22.7	46.1	46.1	18.3	18.3
Park area in municipal/county data but not in commercial data	20.3	NA	21.8	NA	13.5	NA
Park area in commercial data but not in municipal/county data	NA	9.9	NA	74.6	NA	6.3
**Percentage of study area in parks**	2.3	1.8	5.0	8.9	12.3	9.5

Abbreviation: NA, not applicable.

a North Carolina study area comprised Davidson, Davie, Guilford, Forsyth, Randolph, Rockingham, Stokes, Surry, and Yadkin Counties (1,837 sq mi).

b Total number of parks derived from combining verified municipal/county and commercial data.

c Maryland study area comprised 79 zip code areas in Anne Arundel, Carroll, Harford, Howard, and Baltimore counties and Baltimore city (1,351 sq mi).

d New York study area comprised 183 zip code areas in Bronx, Brooklyn, Manhattan, and Queens boroughs and Westchester County (260 sq mi).

e Data from municipal/county government sources collected from 2009 through 2012.

f Data from Esri (Esri, Redlands, California), 2010.

g Parks that were identified in both data sources.

h Includes 28 parks in North Carolina, 55 in Maryland, and 30 in New York that did not meet our definition of a park, which was defined as public place set aside for physical activity and enjoyment. This definition did not include cemeteries, mobile home parks, historic sites, professional stadiums, country clubs, zoos, private parks, private facilities (such as stand-alone baseball or tennis facilities), and stand-alone recreation centers.

i The exact area where parks from municipal/county data were overlaid with parks from the commercial data.

To examine the effect of parks missing from either data source, we explored how the CDC indicator of at least 1 park within a census block or 0.5 miles from the block boundary varied with commercial data and municipal/county data. On the basis of verified (ie, via Internet searches, telephone inquiries, and audits) and combined data sources (ie, parks identified from either or both commercial and municipal/county source), the proportion of census blocks with park access was 35.2% in North Carolina, 64.1% in Maryland, and 97.9% in New York (Table 2). Verified combined parks from municipal/county data sources were more accurate than estimates from commercial sources for North Carolina (absolute proportion difference, 1.1% municipal/county vs 6.2% commercial) and New York (absolute proportion difference, 0.7% municipal/county vs 28.6% commercial), but less accurate for Maryland (absolute proportion difference, 7.6% municipal/county vs 5.2% commercial).

**Table 2:** Parks per Census Block, by Data Source and Study Area: North Carolina, Maryland, and New York, 2009–2012

Study Area	No. of Census Blocks in Area	Municipal/County^a^, n (%)	Commercial^b^, n (%)	Combined, n^c^ (%)
North Carolina	37,492	12,798 (34.1)	10,855 (29.0)	13,214 (35.2)
Maryland	38,356	21,685 (56.5)	22,610 (58.9)	24,598 (64.1)
New York	32,819	31,910 (97.2)	22,741 (69.3)	32,134 (97.9)

a Data from municipal/county government sources collected from 2009 through 2012.

b Data from Esri (Esri, Redlands, California), 2010.

c Data from both municipal/county government and commercial (Esri) sources combined.

To examine the impact of missing parks in either data source, we also quantified the facilities missed if relying only on 1 data source (Table 3). For example, if relying only on municipal/county park data, the data would be missing 12 parks with baseball or softball fields in NC, 30 parks in MD, and 14 in NY. If relying only on the commercial park data, the data file would be missing 34 parks with baseball or softball fields in NC, 72 in MD, and 105 in NY.

**Table 3:** Park Facilities Missed by Relying on 1 Data Source, by Study Area^a^

Parks with Each Facility	Park Facilities Missed if Relying on Municipal/County Data Only^b^			Park Facilities Missed if Relying on Commercial Data Only^b^		
	North Carolina (n = 32) n^a^ (%)	Maryland (n = 65) n^a^ (%)	New York (n = 50) n^a^ (%)	North Carolina (n = 119) n^a^ (%)	Maryland (n = 187) n^a^ (%)	New York (n = 640) n^a^ (%)
**Outdoors**						
Baseball or softball fields	12 (37.5)	30 (46.2)	14 (28.0)	34 (28.6)	72 (38.9)	105 (16.4)
Basketball hoops	5 (15.6)	26 (40.0)	17 (34.0)	25 (21.0)	84 (44.9)	391 (61.2)
Bocce ball courts	0	0	1 (2.0)	0	1 (0.5)	15 (2.3)
Cricket fields	0	0	0	0	0	2 (0.3)
General purpose fields	2 (6.3)	23 (35.4)	5 (10.0)	7 (5.9)	60 (32.1)	19 (3.0)
Golf holes	1 (3.1)	0	1 (2.0)	7 (5.9)	3 (1.6)	4 (0.6)
Football fields	0	1 (1.5)	1 (2.0)	3 (2.5)	0	14 (2.2)
Skate park	0	1 (1.5)	1 (2.0)	4 (3.4)	2 (1.1)	5 (0.2)
Soccer fields	1 (3.1)	0 (0.0)	0	11 (9.2)	2 (1.1)	13 (2.0)
Swimming pools	3 (9.4)	2 (3.1)	2 (4.0)	7 (5.9)	9 (4.8)	23 (3.6)
Tennis courts	7 (21.9)	14 (21.5)	4 (8.0)	17 (14.3)	32 (17.1)	33 (5.2)
Tracks	0	0	1 (2.0)	0	0	12 (1.9)
Volleyball courts	9.4	4 (6.2)	0	9 (7.6)	9 (4.8)	32 (5.0)
**Outdoors or indoors**						
Racquetball, handball, or squash courts	1 (3.1)	0	5 (10.0)	0	1 (0.5)	394 (61.6)
**Indoors**						
General purpose fields	0	0	1 (2.0)	0	2 (1.1)	0
Swimming pools	1 (3.1)	0	0	0	0	7 (1.1)

a Parks missed when relying only on commercial data or only on municipal/county data. The numbers given in this table for “parks missed when relying only on municipal/county data” (32, North Carolina; 65, Maryland; and 50, New York) are lower numbers than those shown in Table 1 (60, 120, 80, respectively). The difference is because some parks in the municipal and county data did not meet the study’s park definition.

b Number and percentage of facilities in missed parks.

## Discussion

When comparing parks data obtained from commercial and municipal/county sources, we found that both data sources omitted parks whose existence was verified through Internet searches, telephone inquiries, or on-the-ground audits. The most accurate park assessment was derived by combining verified commercial and municipal/county data together.

There are several advantages and disadvantages to both commercial data and municipal/county park data for research purposes. Although it may be necessary to purchase commercial data, such data may be easier to use and require less staff time. A disadvantage to commercial data sources is that they may include spaces that are not considered parks by the researchers’ definition.

Municipal/county parks data files were generally more complete than commercial data sources; however, acquiring them required significant staff time. The quality of municipal/county GIS data varied across geographic areas, and it was unclear how frequently data files were updated. Therefore, they may be temporally mismatched across multiple administrative boundaries. Users should be aware that national parks, state parks, and forest areas may not be included in municipal/county parks data.

Neither municipal/county or commercial sources of parks data provided information on facilities in the park or the quality of parks. Facilities offered at the park can be identified, as in our study, through Internet searches, telephone calls, and site visits if needed; data on park quality can be collected through site visits or, as in New York City, through its park inspection program (8). Neither the municipal/county or commercial data sources included private neighborhood parks that may be accessible to the public. Whether these parks are of interest can be determined through an audit or site visit, although private neighborhood parks without road access may still be missed. Audits may miss parks that are unnamed (ie, lack signage), and conducting audits may require significant time and cost (14). Although park shapefiles in commercial data sources are static, we learned that they are fluid in municipal/county sources. By “fluid” we mean that parks may be added, removed, or renamed and that facilities within parks can change over time. Park shapefiles and inventory of amenities should be updated if a study spans an extended period.

### Impact of the park data source

To explore the impact of the 2 park data sources, we used a CDC indicator: the percentage of census blocks that had parks within their block or within 0.5 miles of the boundary (16) (Table 2). We compared our results with CDC’s finding of a 20% median across the United States of access to parks, ranging from 2% (Mississippi) to 47% (California). For its calculations, CDC used national, state, county, and local parks data from a 2007 commercial source. We calculated the indicator by using municipal/county and commercial parks data and found that the result varied between the 2 data sources and across locations. When compared with the combined and verified park data, the absolute prevalence difference ranged from 0.7% to 7.6% for municipal/county data and 5.2% to 28.6% for commercial data. The differences were most remarkable for the commercial data for New York, because a large number of parks were missing. For North Carolina and New York, municipal/county data provided estimates closer to the combined and verified data than did data from the commercial source. However, for Maryland the commercial data provided estimates closer to the combined and verified data because of the larger spatial area of parks that were in the commercial data but not in the municipal/county data.

We also calculated the effect of parks missing from both data sources by quantifying the facilities at each park that were missed (Table 3). We found that parks that were missed did contain a variety of facilities, a finding that had a larger impact on most commonly found active park facilities, such as baseball or softball fields and basketball hoops.

### Study limitations

Our study had several limitations. First, we did not compare results from the 2 data sources used here (ie, park data from the commercial source and data from municipal/county sources) with other commercial data sources that may be available. Second, we were unable to compare results by urbanicity and recognize that the quality of parks data for urban and rural areas may differ. Third, in some instances, the park shapefiles from the 2 data sources did not exactly match. In these situations, we determined if parks from the 2 sources were the same parks or different parks by comparing the park name, shape, and percentage of the area overlapping from visual inspection, by comparing names to see if they matched, and by the percentage of park area that matched.

 This method was not subjective, because we did not go to the parks to see the differences. Fourth, the metadata from both sources could have provided more information on the geospatial data, such as the content, quality, positional accuracy, coverage, scale, and date of collection, but was not provided (17).

### Conclusion

GIS-derived measures of parks allow practitioners and researchers to investigate park accessibility and associations of parks with physical activity by nearby residents. Studies of park accessibility and associations with physical activity would benefit from quantification of the degree of error in GIS data and ultimately the potential bias that such error introduces to surveillance measures and to environment–health associations (18). In assessing both commercial and municipal/county data sources, we found count errors (neither source listed all parks), attribute errors (commercial sources listed some parks that were not verified as such), and positional errors (parks listed in the 2 data sources did not always align). Using both data sources and verifying that parks existed was the most accurate way to develop the park shapefile in this study. However, it is still possible that parks were missed even though we used both sources.

These findings indicate that practitioners and researchers should check park shapefiles from commercial or municipal/county sources before using them by verifying them against other sources of information. A comprehensive parks file for the entire United States, developed using standardized GIS protocols (17,19), could facilitate parks-related research. With more than 9,000 local parks and recreation departments and organizations that manage more than 108,000 public park facilities and 65,000 indoor facilities (20), the coordination of data across jurisdictions is complex. A database to house this information that is regularly updated could be useful to future research and for surveillance purposes.
