# Erratum, Vol. 10, June 6 Release

**DOI:** 10.5888/pcd11.120265e

**Published:** 2014-03-27

In the article “Using Geographic Information Systems to Compare Municipal, County, and Commercial Parks Data,” the author made inadvertent errors in several calculations as a result of overlapping park shape files that were not accounted for in the analysis. This erratum provides corrected results that account for the overlap. The corrections were made to our website on March 27, 2014, and the corrected article appears online at http://www.cdc.gov/pcd/issues/2013/12_0265.htm. The authors regret any confusion or inconvenience these errors may have caused.

The following corrections have been made to the article:

**Results (Abstract)**

Commercial parks data did not include 30.5% (116/380, 20.2 sq mi) of North Carolina, 42.9% (187/436, 21.3 sq mi) of Maryland, and 71.7% (629/881, 12.7 sq mi) of New York parks that we found and verified from municipal/county sources. Municipal/county data did not include 15.8% (60/380, 10.1 sq mi) of North Carolina parks, 27.5% (120/436, 74.8) of Maryland parks, and 9.1% (80/881, 7.8 sq mi) of New York parks that we found and verified from commercial sources.

**Results**

Overall, we verified the existence of 380 parks in the NC study area, 436 parks in the Maryland study area, and 881 parks in the New York study area (Table 1). The commercial data source did not include the following percentage of parks found and verified in municipal/county sources: 30.5% (116/380, 20.2 sq mi) in North Carolina, 42.9% (187/436, 21.3 sq mi) in Maryland, and 71.7% (629/881, 12.7 sq mi) in New York. The municipal/county data sources did not include the following parks found and verified in the commercial source: 15.8% (60/380, 10.1 sq mi) in North Carolina, 27.5% (120/436, 74.8 sq mi) in Maryland, and 9.1% (80/881, 7.8 sq mi) in New York. Municipal/county data sources showed higher percentages of land area with parks for North Carolina and New York than did the commercial data sources but a lower percentage for Maryland.

**Table 1:** Comparison of Parks Data Obtained From Municipal/County Sources with Data Obtained from Commercial Sources in 3 Locations: North Carolina, Maryland, and New York, 2009–2012

Park Details	North Carolina^a^, n = 380^b^		Maryland^c^, n = 436^b^		New York^d^, n = 881^b^	
	Municipal/ County^e^	Commercial^f^	Municipal/ County^e^	Commercial^f^	Municipal/ County^e^	Commercial^f^
**Number of parks**						
Number of parks, total	320	261	316	246	801	251
Parks in both data sources^g^	204	201	129	126	172	171
Parks in municipal/county data but not in commercial data	116	NA	187	NA	629	NA
Parks in commercial data but not in municipal/county data^h^	NA	60	NA	120	NA	80
**Park area (sq mi)**						
Park area, total	42.7	32.5	67.2	120.7	29.5	24.7
Park area spatially overlaid^i^	22.5	22.5	45.9	45.9	16.9	16.9
Park area in municipal/county data but not in commercial data	20.2	NA	21.3	NA	12.7	NA
Park area in commercial data but not in municipal/county data	NA	10.1	NA	74.8	NA	7.8
**Percentage of study area in parks**	2.3	1.8	5.0	8.9	11.4	9.5

Abbreviation: NA, not applicable.

a North Carolina study area comprised Davidson, Davie, Guilford, Forsyth, Randolph, Rockingham, Stokes, Surry, and Yadkin Counties (1,837 sq mi).

b Total number of parks derived from combining verified municipal/county and commercial data.

c Maryland study area comprised 79 zip code areas in Anne Arundel, Carroll, Harford, Howard, and Baltimore counties and Baltimore city (1,351 sq mi).

d New York study area comprised 183 zip code areas in Bronx, Brooklyn, Manhattan, and Queens boroughs and Westchester County (260 sq mi).

e Data from municipal/county government sources collected from 2009 through 2012.

f Data from Esri (Esri, Redlands, California), 2010.

g Parks that were identified in both data sources.

h Includes 28 parks in North Carolina, 55 in Maryland, and 30 in New York that did not meet our definition of a park, which was defined as public place set aside for physical activity and enjoyment. This definition did not include cemeteries, mobile home parks, historic sites, professional stadiums, country clubs, zoos, private parks, private facilities (such as stand-alone baseball or tennis facilities), and stand-alone recreation centers.

i The exact area where parks from municipal/county data were overlaid with parks from the commercial data.

**Table 3:** Park Facilities Missed by Relying on 1 Data Source, by Study Area^a^

Parks with Each Facility	Park Facilities Missed if Relying on Municipal/County Data Only^b^			Park Facilities Missed if Relying on Commercial Data Only^b^		
	North Carolina (n = 32) n^a^ (%)	Maryland (n = 65) n^a^ (%)	New York (n = 49) n^a^ (%)	North Carolina (n = 116) n^a^ (%)	Maryland (n = 187) n^a^ (%)	New York (n = 629) n^a^ (%)
**Outdoors**						
Baseball or softball fields	12 (37.5)	30 (46.2)	13 (26.0)	34 (29.3)	72 (38.9)	104 (16.5)
Basketball hoops	5 (15.6)	26 (40.0)	17 (34.0)	25 (21.6)	84 (44.9)	383 (60.9)
Bocce ball courts	0	0	1 (2.0)	0	1 (0.5)	14 (2.2)
Cricket fields	0	0	0	0	0	2 (0.3)
General purpose fields	2 (6.3)	23 (35.4)	5 (10.0)	7 (6.0)	60 (32.1)	19 (3.0)
Golf holes	1 (3.1)	0	1 (2.0)	7 (6.0)	3 (1.6)	4 (0.6)
Football fields	0	1 (1.5)	1 (2.0)	3 (2.6)	0	14 (2.2)
Skate park	0	1 (1.5)	1 (2.0)	4 (3.4)	2 (1.1)	5 (0.8)
Soccer fields	1 (3.1)	0 (0.0)	0	11 (9.5)	2 (1.1)	13 (2.1)
Swimming pools	3 (9.4)	2 (3.1)	2 (4.0)	7 (6.0)	9 (4.8)	23 (3.7)
Tennis courts	7 (21.9)	14 (21.5)	4 (8.0)	17 (14.7)	32 (17.1)	33 (5.2)
Tracks	0	0	1 (2.0)	0	0	12 (1.9)
Volleyball courts	9.4	4 (6.2)	0	9 (7.8)	9 (4.8)	32 (5.1)
**Outdoors or indoors**						
Racquetball, handball, or squash courts	1 (3.1)	0	5 (10.0)	0	1 (0.5)	387 (61.5)
**Indoors**						
General purpose fields	0	0	1 (2.0)	0	2 (1.1)	0
Swimming pools	1 (3.1)	0	0	0	0	7 (1.1)

a Parks missed when relying only on commercial data or only on municipal/county data. The numbers given in this table for “parks missed when relying only on municipal/county data” (32, North Carolina; 65, Maryland; and 49, New York) are lower numbers than those shown in Table 1 (60, 120, 80, respectively). The difference is because some parks in the municipal and county data did not meet the study’s park definition.

b Number and percentage of facilities in missed parks.

